# Prevalence and predictors of uncommon features in FSHD1 patients: insights from the French FSHD registry

**DOI:** 10.1186/s13023-025-03877-z

**Published:** 2025-09-02

**Authors:** Benoît Sanson, Abderhmane Slioui, Jérémy Garcia, Lori Klouvi, Julie Lejeune, Caroline Stalens, Céline Guien, Sitraka Rabarimeriarijaona, Rafaëlle Bernard, Juliette Nectoux, Sharham Attarian, Anne-Laure Bédat-Millet, Françoise Bouhour, François Constant Boyer, Jean-Baptiste Chanson, Ariane Choumert, Pascal Cintas, Elisa De La Cruz, Léonard Féasson, Maxime Fournier, Karima Ghorab, Agnès Jacquin-Piques, Pascal Laforêt, Armelle Magot, Maud Michaud, Jean-Baptiste Noury, Guilhem Solé, Marco Spinazzi, Tanya Stojkovic, Céline Tard, Luisa Villa, Christophe Béroud, Sabrina Sacconi, the French FSHD registry collaboration group

**Affiliations:** 1 Université Côte d’Azur, Service Système Nerveux Périphérique & Muscle, Centre Hospitalier Universitaire de Nice, Nice, France; 2 Université Caen-Normandie, Pôle des Formations et de Recherche en Santé, Caen, France; 3 Direction des Opérations et Innovations Scientifiques, AFM-Telethon, Évry, France; 4 Genomnis SAS, Marseille, France; 5 APHM, Service de Génétique Médicale, Biogénopôle, CHU Timone Adultes, Marseille, France; 6 Service de Médecine Génomique des Maladies de Système et d’Organe, Hôpital Cochin, AP.HP.CUP, Paris, France; 7 Service des Maladies Neuromusculaires et de la SLA, Hôpital de La Timone, Marseille, France; 8 Centre de Référence des Maladies Neuromusculaires, CHU de Rouen, Rouen, France; 9 Referral Centre for Neuromuscular Disease, Hôpital Neurologique, Lyon-Bron, France; 10 NMD Reference Center, Reims Champagne-Ardenne University Hospital, EA3797, Reims, France; 11 Centre de Référence des Maladies Neuromusculaires, Service de Neurologie, European Reference Network for Neuromuscular Diseases (EURO-NMD), CHU de Strasbourg, Strasbourg, France; 12 Centre de Référence des Maladies Neuromusculaires, CHU de La Réunion, Saint-Pierre, France; 13 Department of Neurology, Toulouse University Hospital, Toulouse, France; 14 Department of Neurology, Montpellier University Hospital Center, Gui de Chauliac Hospital, Montpellier, France; 15 Centre de Référence des Maladies Neuromusculaires, CHU de Saint-Étienne, Saint-Étienne, France; 16 Centre de Référence des Maladies Neuromusculaires, CHU de Caen, Caen, France; 17 Centre de Référence des Maladies Neuromusculaires, CHU de Limoges, Limoges, France; 18 Department of Neurology, Clinical Neurophysiology Unit, Centre de Compétence des Maladies Neuromusculaires, CHU Dijon Bourgogne, Dijon, France; 19 APHP, Centre de Référence des Maladies Neuromusculaires, Hôpital de Garches, Garches, France; 20 Referral Center for Neuromuscular Diseases AOC, CHU Nantes, Nantes, France; 21 Centre de Référence des Maladies Neuromusculaires, CHRU Nancy, Nancy, France; 22 Centre de Référence des Maladies Neuromusculaires, CHU de Brest, Brest, France; 23 Centre de Référence des Maladies Neuromusculaires AOC, FILNEMUS, EURO-NMD, Hôpital Pellegrin, CHU de Bordeaux, Bordeaux, France; 24 Centre de Référence des Maladies Neuromusculaires AOC, FILNEMUS, Service de Neurologie, CHU d’Angers, Angers, France; 25 APHP, Centre de Référence des Maladies Neuromusculaires Nord/Est/Île-de-France, Institut de Myologie, Sorbonne Université, Hôpital Pitié-Salpêtrière, Paris, France; 26 U1172, Centre de Référence des Maladies Neuromusculaires Nord/Est/Île-de-France, CHU de Lille, Lille, France; 27 Aix Marseille Univ, INSERM, INRAE, C2VN, Marseille, France; 28 Institute for Research on Cancer and Aging of Nice (IRCAN), INSERM U1081, CNRS UMR 7284, Université Côte d’Azur (UCA), Faculté de Médecine, Nice, France

**Keywords:** Facioscapulohumeral dystrophy (FSHD), FSHD1, French FSHD registry, Uncommon phenotypes, Clinical heterogeneity, Borderline D4Z4 repeat numbers, Age of onset

## Abstract

**Background:**

Facioscapulohumeral muscular dystrophy (FSHD) is characterized by a typical pattern of muscle involvement, yet it encompasses a wide spectrum of phenotypes, including less common features that remain incompletely defined in the literature. While previous studies have highlighted this clinical variability, no consensus has been reached on how to classify uncommon manifestations, nor have specific predictors been identified. This study aims to describe these uncommon features and explore potential predictors, utilizing data from the French FSHD registry. To this end, we analysed data from 306 FSHD1 patients across nine French neuromuscular referral centres. Descriptive statistics, univariate analyses, and multiple logistic regression models were employed to examine uncommon characteristics and their predictors.

**Results:**

Uncommon features were observed in 19.6% of cases. The most common was a discrepancy between disease severity and D4Z4 repeat unit (RU) count (41.7%), followed by predominant impairment at proximal lower limb or distal upper limb muscles (21.7%). Three unanticipated features emerged: isolated or predominant axial impairment, anosmia and atopic dermatitis. Univariate analysis revealed that uncommon features were associated with higher RU count (6.5 ± 2.1 vs. 5.8 ± 1.8 in typical patients) and older age of onset (32.0 ± 18.8 years vs. 25.0 ± 15.4 years). Such features were more prevalent in the borderline 8–10 RU range, an association confirmed by multivariate analysis (OR = 2.43, 95% CI 1.21 to 4.87). Later age of onset consistently emerged as a factor across multiple multivariate models.

**Conclusions:**

This study documents uncommon FSHD features, revealing their association with the 8–10 RU range and later age of onset. These findings further support a complex interplay among genetic and epigenetic modifiers and ageing in shaping the clinical phenotype of FSHD, especially in patients carrying borderline D4Z4 arrays. Differential phenotypes, particularly in relation to RU range and age of onset, points to the importance of harmonized, comprehensive clinical and genetic assessments. Recognizing uncommon features may improve diagnostic accuracy and guide individualized management strategies, highlighting the need for tailored approaches to patient care.

## Background

Facioscapulohumeral dystrophy (FSHD, OMIM #158900) is the third most frequent hereditary myopathy [1]. While no epidemiological study has been performed in France, prevalence estimates from the United States, The Netherlands and Italy, ranging from 1:20,000 to 1:8000, suggest that at least 3500 persons are affected in the country [2–4].

The phenotypic expression and disease progression in FSHD are highly variable, even within families, yet typically follow a characteristic pattern. Symptoms usually first appear asymmetrically in the late twenties, predominantly affecting the upper girdle and face, then gradually progressing to the trunk, lower girdle and lower limbs [5]. Early involvement of leg muscles is also observed, with some patients presenting with distal weakness, often manifesting as foot drop. Nearly 20% of patients eventually require wheelchair assistance [6]. In severe cases, extramuscular complications may occur, including central nervous system, ophthalmic, and auditory impairments [7].

The primary cause of FSHD is the truncation of the D4Z4 macrosatellite repeat array in the subtelomeric region of chromosome 4, leading to chromatin relaxation and derepression of the DUX4 protein, which is toxic for the muscle cell [8]. FSHD1, comprising over 95% of cases, is characterized by 1–10 repeat units (RU), on a permissive 4qA haplotype, while the rarer FSHD2 form has been ascribed to mutations in D4Z4 chromatin repressors, primarily *SMCHD1* [9]. FSHD is increasingly viewed as a continuum between these two types [10], with ongoing debate about the adequacy of current diagnostic tests for borderline RU counts (8–10) [10, 11]. Interestingly, patients carrying both an FSHD1 permissive allele and a mutation in the FSHD2-associated gene *SMCHD1* exhibit a more severe phenotype compared to those with only an FSHD1 allele in the borderline RU range [10].

Uncommon presentations, defined in part as the shortage of prototypical features commonly observed in FSHD patients, are increasingly recognized as significant contributors to diagnostic errors and phenotypic variability [12]. Despite their relative frequency, they remain inadequately described, with no consensus on their classification. A clustering analysis on 222 patients from the UK FSHD registry identified four phenotypes: classical FSHD (74%) and three facial-sparing variants characterized by unique patterns of muscle involvement and rate of progression [13]. Ricci et al. developed the Comprehensive Clinical Evaluation Form (CCEF), which categorizes patients into four groups (A, B, C, D) based on facial involvement, pattern and severity of muscle impairment, and presence of predefined uncommon features [14].

Analysis on a cohort of 187 Italian registry patients with 7–8 RU revealed that 26.7% presented uncommon features and were classified as category D, while 19.3% exhibited mild forms without such features and were grouped in category B [15]. A similar study involving 134 Italian registry patients with 9–10 RU reported 18.7% in category D [16]. No data have been published regarding patients carrying 1–6 RU. Furthermore, the CCEF and associated studies do not provide a detailed characterization of uncommon features encountered.

The French FSHD registry, which uniquely combines self-reported patient data with clinical evaluations from neuromuscular specialists, offers a valuable resource for exploring uncommon features [17, 18]. The registry employs a comprehensive definition of such features, which includes the discrepancy between D4Z4 RU count and disease severity, evidence for another neuromuscular disease and a free-text field for neuromuscular specialists to report any relevant unusual feature.

Here we report the spectrum of uncommon features documented in the French registry, drawing from a cohort of 306 patients. We analyse variables associated with these uncommon characteristics, focusing on key factors of disease severity, such as age of onset and D4Z4 RU count [19]. By exploring these relationships, we aim to shed light on the complex determinants of clinical heterogeneity in FSHD.

## Patients and methods

### Patient cohort

**Fig. 1:**
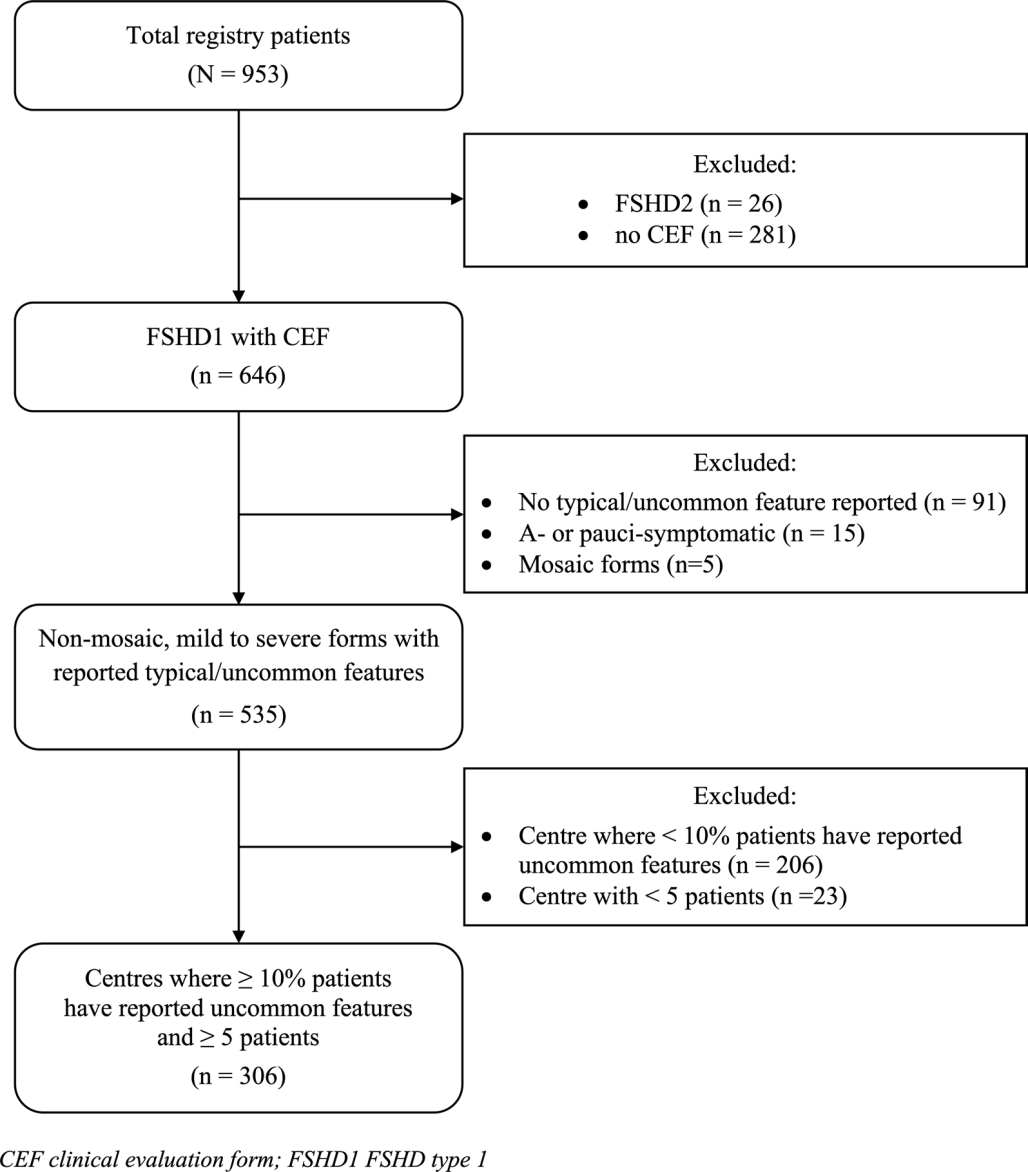
Patient selection process

The study utilized retrospective data from the French FSHD registry [17, 18], a comprehensive database designed to capture detailed phenotypic and genetic data on FSHD patients in France. Accessible at www.fshd.fr, the registry includes both self-reported information from patients and clinical evaluations from neuromuscular specialists at referral centres nationwide. This analysis focused exclusively on FSHD1 patients with documented typical or uncommon features, as recorded in a dedicated phenotypic classification section of the clinical evaluation form (available at https://fshd.fr/documents), excluding asymptomatic and mosaic cases (Fig. 1). To ensure data quality, we excluded referral centres that had completed the classification section for fewer than 50% of their patients, or reported fewer than 10% of cases with uncommon features, while acknowledging a potential overestimation of their prevalence (see Data homogeneity subsection). Additionally, only centres with at least five patients were considered for inclusion.

From an initial pool of 953 patients, we identified 646 FSHD1 patients with available clinical evaluation. After applying exclusion criteria, data from 306 patients across nine neuromuscular referral centres (Bordeaux, La Réunion, Limoges, Lyon, Montpellier, Nantes, Nice, Reims and Strasbourg) were included in the analysis. In cases with multiple CEFs per patient, the most recent form documenting typical or uncommon features was used.

### Molecular diagnosis

To assess diagnostic validity, we examined 4qA haplotyping and inheritance data. Confirmation of the 4qA allele was available in 25.8% of cases (n = 79), reflecting limited access to the required technology. Autosomal dominant inheritance was documented in 74% of cases (n = 168). Importantly, only one patient has been excluded from the registry because of a 4qB haplotype, indicating a very low risk of including false-positive FSHD1 diagnoses. Extended genetic testing—such as *SMCHD1* sequencing or methylation analysis—was not routinely performed in registry patients.

Thirteen patients with an unknown D4Z4 repeat size, all diagnosed by trained neuromuscular specialists, were included based on supporting evidence, primarily a documented familial history of FSHD (n = 10). Molecular data for 10 of these patients became available only after the completion of the analyses presented here.

### Data collection and variables

Clinical evaluation was recorded using a clinical evaluation form (CEF), filled out by specialized physicians, which gathers extensive information on demographic details, clinical symptoms, genetic data, and systemic impairments. Variable selection was guided by clinical judgment. Key variables analysed included age, weight, height, age of onset, typical and uncommon FSHD features (for a full list and results, see Table 3 in the Results section), presence of systemic impairments, and disease severity assessed using a modified Clinical Severity Scale (CSS) [20]. The CSS employed grades twice those outlined by Ricci et al., with 0 indicating the absence of symptoms. Patients scoring 6–10 were classified as more severely affected; those scoring 1–5 as having a milder form. Uncommon features were recorded using a predefined checklist complemented by a free-text field to document any relevant observations. The checklist was developed through a thorough literature review and consensus from an FSHD clinical expert panel, with plans for periodic updates.

### Statistical analysis

Data analysis was conducted using SAS software (Version 9.4, SAS Institute Inc., Cary, NC, USA). Statistical significance was set at *p* < 0.05 for all analyses. Graphs were created using the matplotlib library in Python 3.

We conducted a descriptive analysis to outline the cohort’s demographic, clinical, and genetic characteristics. Continuous variables were summarized using means and standard deviations, while categorical variables were described as frequencies and percentages. To assess homogeneity between typical and uncommon subgroups, we applied the Chi-square test for categorical variables, the Student’s t test for two-group comparisons of quantitative variables, ANOVA for multi-group comparisons, and the Armitage test for ordinal variables.

We then explored potential relationships between uncommon features and explanatory covariates through multiple logistic regression models. For each model, we calculated odds ratios (OR) with 95% confidence intervals (CI) and associated *p*-values for each covariate included.

Disease duration was computed as the difference between age at assessment and age of onset (in years). In addition, we stratified the cohort based on the number of systemic impairments, RU count (low: 2–4; medium: 5–7; high: 8–10) and CSS (less severe: < 6 vs more severe: ≥ 6). Analyses were conducted using either discrete or stratified RU count, as appropriate.

### Data homogeneity

To ensure the dataset was homogeneous, we assessed the distribution of typical and uncommon forms across participating sites. Centres reporting less than 10% uncommon cases were excluded to mitigate potential underreporting biases. This threshold was established to balance the exclusion of low-reporting centres with maintaining a sufficient sample size. While somewhat arbitrary, it was informed by previously reported uncommon prevalence rates of 26% in the UK registry [13] and 18–27% in the Italian registry [15, 16], as well as clinical experience. Furthermore, centres with fewer than five patients were excluded to provide reliable estimates of uncommon case percentages. This criterion was set to reduce the influence of random variation in small samples and ensures a more representative basis for identifying uncommon patterns. This approach aimed to guarantee a representative sample while maintaining data quality. Nonetheless, the registry was not designed to support inter-rater reliability analysis, and lacks contextual information in the phenotypic classification section, which precluded any assessment of site-specific variation in how uncommon features were interpreted or recorded.

### Ethical considerations

The study was conducted in accordance with ethical standards. The French FSHD registry received initial approval from CNIL (authorization number 912291) and CCTIRS (favourable opinion number 12.004bis). Renewed approvals were obtained in 2020 (CNIL authorization number 2217209) to comply with updated European and French data protection regulations. All patients provided written consent for their data to be used in the registry.

## Results

### Description of the cohort

**Fig. 2:**
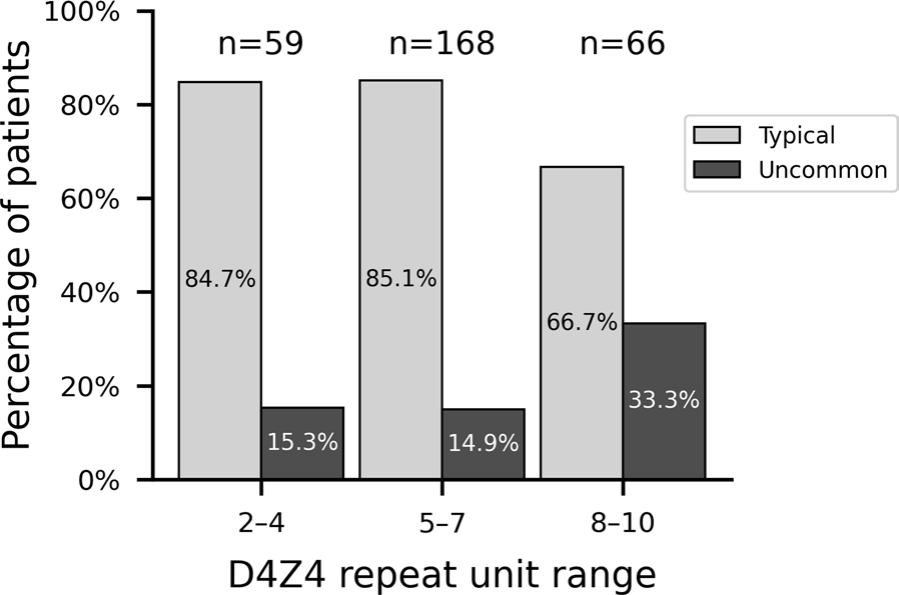
Proportion of patients with typical and uncommon features by D4Z4 repeat unit (RU) count strata. Cases with unknown RU count (n = 13) are not shown

The cohort comprised 173 males (56.5%) and 133 females (43.5%), with a mean age of 52.7 ± 16.5 years (Table 1). The mean D4Z4 RU count was 6.0 ± 1.8, distributed as follows: 2–4 (20.1%), 5–7 (57.3%), and 8–10 (22.5%). Typical FSHD characteristics were observed in 246 patients (80.4%), while 60 patients (19.6%) exhibited uncommon features. These proportions were consistent across all sites (Table 2; *p* = 0.606). The mean D4Z4 RU count for typical and uncommon forms was 5.8 ± 1.8 and 6.5 ± 2.1, respectively, with their distribution across RU count strata shown in Fig. 2.

**Table 1:** Descriptive statistics of the cohort

Characteristic	n = 306
Gender	
Male	173 (56.5%)
Female	133 (43.5%)
D4Z4 RU count*	
n (missing)	293 (13)
Mean ± SD	6.0 ± 1.8
2**–**4	59 (20.1%)
5**–**7	168 (57.3%)
8**–**10	66 (22.5%)
Age (years)	
Mean ± SD	52.7 ± 16.5
Range	14.0–84.8
Age of onset (years)	
n (missing)	276 (30)
Mean ± SD	26.3 ± 16.3
Range	1.0–73.0
FSHD form	
Typical	246 (80.4%)
Uncommon	60 (19.6%)

SD standard deviation; RU repeat unit; *The total percentage of strata does not equal 100% because of rounding

**Table 2:** FSHD form distribution by referral centre

Sites	Typical patients	Patients with uncommon features	Total	*p*-value
Nice	82 (79.6%)	21 (20.4%)	103	
Bordeaux	49 (86.0%)	8 (14.0%)	57	
Strasbourg	30 (85.7%)	5 (14.3%)	35	
Montpellier	25 (78.1%)	7 (21.9%)	32	
Nantes	16 (66.7%)	8 (33.3%)	24	
Limoges	15 (71.4%)	6 (28.6%)	21	
Lyon	12 (85.7%)	2 (14.3%)	14	
Reims	12 (85.7%)	2 (14.3%)	14	
La Réunion	5 (83.3%)	1 (16.7%)	6	
Total	246 (80.4%)	60 (19.6%)	306	0.606

### Distribution of uncommon features

The most prevalent uncommon feature, observed in 41.7% of patients exhibiting such features (n = 25), was a discrepancy between disease severity and D4Z4 RU count (Table 3). Specifically, this refers to patients who displayed unexpectedly mild phenotypes despite having low RU counts (n = 10, 2–5 RU), or conversely, severe phenotypes despite higher RU counts (n = 14, 7–10 RU); RU count was unknown for one patient. Predominant impairment at proximal lower limbs or distal upper limbs was the second most frequent uncommon presentation (21.7%).

**Table 3:** Distribution of uncommon features reported in the cohort

Feature	n = 60
Discrepancy between FSHD severity and D4Z4 RU count	25 (41.7%)
Predominant impairment at proximal lower limbs or distal upper limbs	13 (21.7%)
Other neuromuscular disease	6 (10.0%)
Electromyographic evidence of neurogenic disease	6 (10.0%)
Primary cardiac or respiratory impairment	5 (8.3%)
Muscle biopsy findings suggestive of another neuromuscular disease	2 (3.3%)
Ocular ptosis and/or diplopia	2 (3.3%)
Unlisted features	20 (33.3%)
Anosmia	8 (13.3%)
Isolated or predominant axial symptoms, including camptocormia	4 (6.7%)
Atopic dermatitis	3 (5.0%)
Other comorbidities	8 (13.3%)
Alzheimer's disease	1 (1.7%)
Behçet syndrome	1 (1.7%)
Cognitive deficit	1 (1.7%)
Erectile dysfunction	1 (1.7%)
Interstitial pneumonia	1 (1.7%)
Laryngeal stridor	1 (1.7%)
Spleen lymphoma	1 (1.7%)
Tremor	1 (1.7%)

RU repeat unit; Patients may present with multiple uncommon features

Electromyographic evidence of neurogenic disease was found in 10.0%, including two cases of radiculopathy and one of diabetic neuropathy; the remaining were unspecified. Other notable features included another neuromuscular disease (10.0%) and primary cardiac or respiratory impairment (8.3%). Less frequent manifestations were ocular ptosis or diplopia (3.3%) and muscle biopsy findings suggestive of another neuromuscular disease (3.3%), with one showing mitochondrial anomalies and the other unspecified.

Additionally, 33.3% presented with unlisted uncommon features, making it the second most frequently reported item overall. Notable among these were anosmia (13.3%), isolated or predominant axial symptoms such as camptocormia (6.7%), and atopic dermatitis (5.0%). Investigators also reported other comorbidities in 13.3%.

Some patients exhibited multiple uncommon features, so percentages sum to more than 100%. Nineteen had two features, comprising nine with two prelisted features (five of whom were reported to show electromyographic evidence of neurogenic disease and another neuromuscular disease); five with one prelisted feature and one unlisted feature (excluding other comorbidities); five with one prelisted and one other comorbidity; three with two other comorbidities.

### Univariate analysis of uncommon features

Univariate analysis revealed significant differences between typical FSHD patients and those with uncommon features (Table 4). The D4Z4 RU count distribution differed markedly between groups (*p* = 0.004). Patients with 8–10 RU exhibited a higher prevalence of uncommon features (33.3%, *p* < 0.001) compared to the overall cohort, while those with 5–7 RU showed a lower prevalence (14.9%, *p* = 0.033). In contrast, no difference was observed for patients with 2–4 RU (15.3%, *p* = 0.399).

**Table 4:** Univariate analysis of uncommon features

Variable	Typical patients	Patients with uncommon features	Total	*p*-value
Sex				0.236
Male	135 (78.0%)	38 (22.0%)	173 (100.0%)	
Female	111 (83.5%)	22 (16.5%)	133 (100.0%)	
D4Z4 RU count				
n (missing)	237 (9)	56 (4)	293 (13)	0.004
Mean (SD)	5.8 (1.8)	6.5 (2.1)	6.0 (1.8)	
2–4	50 (84.7%)	9 (15.3%)	59 (100.0%)	0.399*
5–7	143 (85.1%)	25 (14.9%)	168 (100.0%)	0.033*
8–10	44 (66.7%)	22 (33.3%)	66 (100.0%)	< .001*
Age at assessment				
n (missing)	246 (0)	60 (0)	306 (0)	< .001
Mean (SD)	51.1 (16.1)	59.4 (16.6)	52.7 (16.5)	
Age categories				0.039
10–20	7 (0%)	0 (0%)	7 (100.0%)	
20–50	100 (86.2%)	16 (13.8%)	116 (100.0%)	
> 50	139 (76.0%)	44 (24.0%)	183 (100.0%)	
Age of onset				
n (missing)	224 (22)	52 (8)	276 (30)	0.014
Mean (SD)	25.0 (15.4)	32.0 (18.8)	26.3 (16.3)	
Age of onset categories				0.012
< 10	27 (90.0%)	3 (10.0%)	30 (100.0%)	
10–20	80 (83.3%)	16 (16.7%)	96 (100.0%)	
20–50	104 (81.9%)	23 (18.1%)	127 (100.0%)	
> 50	13 (56.5%)	10 (43.5%)	23 (100.0%)	
Site of first symptoms				
n (missing)	236 (10)	57 (3)	293 (13)	< .001
Facial muscles	41 (91.1%)	4 (8.9%)	45 (100.0%)	
Proximal upper limb	144 (84.2%)	27 (15.8%)	171 (100.0%)	
Distal upper limb	3 (60.0%)	2 (40.0%)	5 (100.0%)	
Proximal lower limb	11 (55.0%)	9 (45.0%)	20 (100.0%)	
Distal lower limb	26 (83.9%)	5 (16.1%)	31 (100.0%)	
Other	11 (52.4%)	10 (47.6%)	21 (100.0%)	
Diabetes				
n (missing)	246 (0)	60 (0)	306 (0)	0.005
Presence	12 (57.1%)	9 (42.9%)	21 (100.0%)	

SD standard deviation; RU repeat unit; *p-value computed by strata

For categorical variables, percentages indicate the proportion of typical patients and of those with uncommon features; for continuous variables, mean values are presented

Patients with uncommon features were significantly older at assessment (59.4 ± 16.6 years vs. 51.1 ± 16.1 years, *p* < 0.001) and accounted for 24% of patients over 50 years of age (*p* = 0.039). Furthermore, these patients experienced later symptom onset (32.0 ± 18.8 years vs. 25.0 ± 15.4 years, *p* = 0.014). Remarkably, 43.5% of patients with symptom onset after age 50 presented uncommon features (*p* = 0.012).

The distribution of initial symptoms differed significantly between typical patients and those with uncommon features (*p* < 0.001). Compared to the whole cohort, uncommon manifestations were more frequent when the first symptoms appeared in the proximal lower limb (45.0%), distal upper limb (40.0%), or were classified as 'other' (47.6%), primarily comprising pain and fatigue (data not shown). Notably, among the 10 patients with pain or fatigue as presenting symptoms, three (30.0%) had uncommon features, representing 5% of all patients with uncommon features, while seven (70.0%) were considered typical, representing 2.7% of all typical patients. Conversely, typical presentations were more prevalent when initial symptoms involved facial (91.1%), proximal upper limb (84.2%), or distal lower limb (83.9%) muscles. Univariate analyses revealed no notable association between uncommon features and severity. However, regarding systemic impairments, patients with diabetes were more likely to display uncommon traits (42.9%, *p* = 0.005), while no other systemic conditions showed a significant relationship.

### Multivariate analysis of uncommon features

**Fig. 3:**
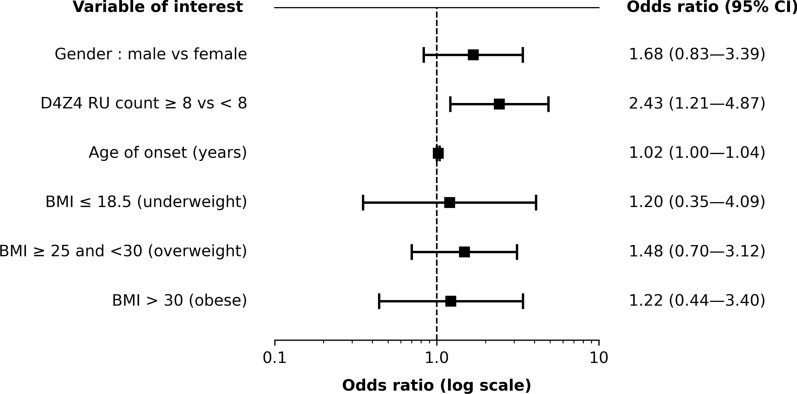
Forest plot of multiple logistic regression for uncommon features adjusted for gender, D4Z4 RU count, age of onset and BMI

D4Z4 RU count emerged as a significant predictor of uncommon features in multiple logistic regression analysis (Table 5 and Fig. 3). Patients with 8–10 RU were significantly more likely to exhibit uncommon features compared to those with fewer RU (OR = 2.43, 95% CI: 1.21 to 4.87, *p* = 0.012). Additionally, multivariate models tested to assess the influence of age of onset revealed a consistent trend: patients who experienced later disease onset showed a slightly higher likelihood of displaying uncommon features (OR = 1.01–1.07; Table 6). Other factors, including gender and BMI, showed no significant association.

**Table 5:** Multiple logistic regression of uncommon features adjusted for gender, D4Z4 RU count, age of onset and BMI

Variable	OR	95% CI	*p*-value
Gender			
Male vs female	1.68	[0.83–3.39]	0.147
D4Z4 RU count by category			
< 8	2.43	[1.21–4.87]	0.012
≥ 8*	*–*	*–*	*–*
Age of onset (years)	1.02	[1.00–1.04]	0.096
BMI (kg/m^2^)			
< 18.5	1.20	[0.35–4.09]	0.766
18.5**–**25*	*–*	*–*	*–*
25–30	1.48	[0.70–3.12]	0.299
> 30	1.22	[0.44–3.40]	0.703

BMI body mass index; CI confidence interval; OR odds ratio; RU repeat unit

^*^Reference category for odds ratio comparisons

**Table 6:** Adjusted odds ratios for age of onset from multiple logistic regression models of uncommon features

Model	Fourth variable	OR for age of onset	95% CI	*p*-value
1	Disease duration	1.07	[1.02–1.11]	0.002
2	Walking ability	1.03	[1.01–1.05]	0.008
3	Clinical Severity Score	1.03	[1.01–1.05]	0.005
4	Cardiac impairment	1.03	[1.01–1.05]	0.006
5	Respiratory impairment	1.03	[1.01–1.05]	0.002
6	Ophthalmic impairment	1.03	[1.01–1.05]	0.011
7	Metabolic impairment	1.03	[1.01–1.04]	0.008
8	GI impairment	1.03	[1.01–1.05]	0.003
9	Endocrine impairment	1.02	[1.01–1.04]	0.010
10	Nb. of systemic impairments	1.02	[1.01–1.04]	0.012

CI confidence interval; OR odds ratio

All models were adjusted for gender, age of onset, BMI category, and a fourth variable. Only models where age of onset showed a significant association (*p* < 0.05) are presented.

## Discussion

### Prevalence of uncommon features

Uncommon features were present in one out of five patients, highlighting the high prevalence of non-classical FSHD, and in line with earlier reports (18–27%) [13, 15, 16]. Difference in classification criteria across national registries likely contribute to discrepancies. For instance, facial-sparing phenotypes are considered uncommon in the UK—likely explaining a lower prevalence in our cohort—classified separately in Italy, and labelled as poorly symptomatic in France. Standardizing definitions across registries would enable more accurate comparisons and enhance understanding of these presentations. Besides, the Italian registry classification tool can serve as a reference framework. In particular, a comparison between the Italian and French classification criteria could be valuable to determine whether patients with uncommon features are consistently identified.

### Variability in muscle involvement pattern and main uncommon features

Nearly 10% of the cohort (25 of 306) exhibited a marked mismatch between D4Z4 RU count and disease severity, underscoring the weak genotype–phenotype correlations in FSHD. Our results support prior studies showing only a moderate inverse relationship between RU count and severity, which reinforces the view that other factors, such as epigenetic alterations, genetic modifiers and age, significantly influence disease expression and deserve further investigation [21].

Predominant impairment at proximal lower limbs or distal upper limbs was the second most frequent uncommon feature. Isolated or predominant axial symptoms, including camptocormia, were reported via free-text fields, and point to a potentially under-recognized manifestation of FSHD, which supports its integration into future registry updates. Together, these findings showcase the variability in muscle involvement patterns in FSHD [21, 22]. While the relationship between FSHD and camptocormia has been recognized for some time [23, 24], it remains scarcely documented. MRI studies suggest that hip extensor muscles contribute to this condition [12], indicating a complex interaction between axial and lower limb muscle involvement. Thus, the presence of camptocormia may blur the distinction between axial, lower limb, and upper limb symptoms, highlighting phenotypic variability, and thereby calling for comprehensive clinical assessment to prevent misdiagnosis.

The array of uncommon features reported here highlights the importance of maintaining a high index of suspicion for FSHD, even in cases with non-classical presentations. This may aid in improving diagnostic accuracy and reaffirms the critical role of genetic testing in confirming diagnosis. While our study did not establish a direct link between uncommon manifestations and disease severity, it suggests that future research should explore specific muscle involvement patterns and their potential correlations with disease progression and prognosis.

### Unexpected manifestations: anosmia, atopic dermatitis

Two unanticipated features emerged from the free-text responses, namely anosmia and atopic dermatitis (AD). Both were notably identified in patients with 8–10 D4Z4 RU, a range where FSHD1 and FSHD2 overlap [10]. Anosmia, previously linked to FSHD2 [25] and Bosma arhinia microphtalmia syndrome [26], may reflect overlapping pathogenic mechanisms. Similarly, AD could suggest a shared epigenetic dysregulation affecting skin barrier function or immune regulation [27–29].

We cannot exclude that these uncommon features arise from other genetic or environmental modifiers. However, importantly, all anosmia data were collected before the COVID-19 pandemic, ruling out infection as a cause [30]. In addition, it is worth noting that the prevalence of anosmia increases with age [31], while that of AD decreases [32]. A systematic, study is warranted to accurately determine the prevalence of anosmia and AD in the FSHD population. Moreover, whole-genome sequencing in relevant subgroups could help identify the genetic modifiers through common variants, with important diagnostic and therapeutic implications. If confirmed, anosmia and AD may expand the clinical spectrum of FSHD, and support further an overlap between FSHD1 and FSHD2, highlighting the complex genetic and epigenetic interplay shaping the phenotype.

### Comorbidities and isolated findings

A higher occurrence of outstanding comorbidities, including other neuromuscular diseases, in the 8–10 RU range, further supports the hypothesis that non-classical features are concentrated in this range [33]. Although most uncommon presentations were also observed in patients with 5–7 RUs, that subgroup was nearly three times larger, making the relative frequency of such features higher in the 8–10 RU range. Nevertheless, this suggests that the overlap between FSHD2 and FSHD1 may extend into shorter RU lengths. A systematic investigation of these features is necessary to confirm it.

Some features, such as tremor and erectile dysfunction, likely reflect coincidental or age-related events. Given their isolated nature and lack of consistent reporting, they are of limited interpretive value and were not discussed in detail. In contrast, the presence of other neuromuscular diseases among cases with uncommon features supports the hypothesis of clinical overlap, particularly in the borderline 8–10 RU range [33].

### Predictors of uncommon features

D4Z4 RU count emerged as a key predictor of uncommon FSHD manifestations, particularly in the borderline 8–10 RU range. Patients in this range often exhibit milder or non-classical symptoms, such as the well-documented facial-sparing phenotype [34–37], which may overlap with those of FSHD2 or other neuromuscular genetic diseases, complicating genotype–phenotype correlations [38].

Our study demonstrates that FSHD patients with uncommon features tend to experience later symptom onset compared to those with classic phenotypes. Data from the Italian FSHD registry show that patients with 7–10 RU [15, 16] typically develop symptoms in their thirties, later than the broader FSHD population, suggesting that the clinical phenotype in this range may depend on additional modifiers including age, comorbidities or coexisting neuromuscular conditions.

Although early onset is generally associated with greater severity [39], our findings highlight a more complex scenario, where severity does not correlate with D4Z4 array size as much as its hypomethylation, especially in the 6–10 RU range [21, 40]. Our data indicate that uncommon phenotypes encompass both mild and severe cases. To fully explore the relationship between disease severity and uncommon presentations, further research is needed, which requires a larger cohort, distinguishing between subgroups, particularly those with unexpectedly mild or severe forms, from other presentations.

Our results underscore the diagnostic challenges posed by phenotypic variability and emphasize the value of standardized classification criteria across national registries. Better harmonization will facilitate cross-cohort comparisons and improve diagnostic accuracy. Furthermore, refining our understanding of FSHD subtypes is critical in light of emerging therapies [41]. Looking ahead, future efforts should leverage registry data and artificial intelligence tools [42] to build predictive models for disease progression [43] and treatment response. Such an approach could enable earlier identification of non-canonical cases and support precision medicine strategies tailored to distinct FSHD subgroups.

### Limitations

Our analysis included only 306 of 953 registry patients, primarily due to incomplete reporting of classification features, which may limit generalizability. Missing data varied across referral centres and items, representing a key constraint. The registry’s focus on adult patients at time of data extraction may also have introduced selection bias. Furthermore, multiple comparisons increase the likelihood of false-positive findings. Future efforts should aim to reduce missing data and apply robust methods—such as multiple imputation or sensitivity analyses—to address it. As the registry grows, statistical power will improve, and criteria like the 10% site inclusion threshold for uncommon cases may be reconsidered.

## Conclusions

This study reports a high prevalence (19.6%) of non-classical FSHD presentations in the French registry, consistent with earlier reports. Key features—such as severity–D4Z4 RU count mismatch and axial symptom predominance—highlight the significant clinical heterogeneity within FSHD. These features were most associated with the borderline D4Z4 repeat range (8–10 RU) and later age of onset, supporting the concept of a phenotypic continuum between FSHD1 and FSHD2. We identified potentially novel features such as anosmia and atopic dermatitis, both emerging notably in the 8–10 RU range. These findings highlight the need to expand the clinical spectrum of FSHD and investigate underlying genetic, epigenetic, and age-related modifiers.

## Declarations

## Ethics approval and consent to participate

The registry was initially approved in 2012 by the French data protection authority (CNIL; authorization number 912291) and the French advisory committee on data processing in health research (CCTIRS; favourable opinion number 12.004bis). It was further approved by CNIL (authorization number 2217209) in 2020 to comply with the revised European and French legislations on data protection and structural changes made in the registry.

## Consent for publication

All patients provided written informed consent for their data to be stored and used in the French FSHD registry.

## Conflict of interests

The authors declare that they have no competing interests.

## Data Availability

Data are accessible on the registry website, www.fshd.fr, through log-in credentials for protecting personal privacy and proprietary information. However, the aggregated data supporting the findings of this study can be obtained upon reasonable request to the French FSHD registry steering committee.

## Abbreviations

AD: Atopic dermatitis
AFM-Téléthon: French Muscular Dystrophy Association
ANOVA: Analysis of variance
CCTIRS: French advisory committee on data processing in health research
CEF: Clinical evaluation form
CI: Confidence interval
CNIL: French data protection agency
CSS: Clinical severity score
FSHD: Facioscapulohumeral muscular dystrophy
OR: Odds ratio
RU: Repeat unit
*SMCHD1*: Structural maintenance of chromosomes flexible hinge domain containing 1
